# Research on Practical Intelligent Mode of Digital Image Economy Based on Improved Genetic Multilayer Neural Network

**DOI:** 10.1155/2021/3113584

**Published:** 2021-11-19

**Authors:** Min Guo, Liying Song, Muhammad Ilyas

**Affiliations:** ^1^School of Economics and Finance, Xi'an Jiaotong University, Xian, Shaanxi 710061, China; ^2^School of Business, Xi'an International University, Xian, Shaanxi 710077, China

## Abstract

In the context of economic globalization and digitization, the current financial field is in an unprecedented complex situation. The methods and means to deal with this complexity are developing towards image intelligence. This paper takes financial prediction as the starting point, selects the artificial neural network in the intelligent algorithm and optimizes the algorithm, forecasts through the improved multilayer neural network, and compares it with the traditional neural network. Through comparison, it is found that the prediction success rate of the improved genetic multilayer neural network increases with the increase of the dimension of the input image data. This shows that, by adding more technical indicators as the input of the combined network, the prediction efficiency of the improved genetic multilayer neural network can be further improved and the advantage of computing speed can be maintained.

## 1. Introduction

Deep learning is a part of machine learning, and machine learning is a part of AI. In other words, there are many patterns or models in machine learning, and in-depth learning is only one branch. It focuses on artificial neural network, and its formation originates from the physical phenomenon of signal interaction between brain neurons. In today's rapidly developing information age, digital image, as a common and effective information carrier, has penetrated every corner of social life, resulting in our increasing demand for image processing. At the same time, in the era of big data, the speed and scale of digital image generation are also very amazing. Therefore, image information processing tasks are required to have the characteristics of high efficiency, high performance, and intelligence. Feature expression is the key of image processing. The traditional feature design needs to be completed manually, but this method is complex and has high requirements for the designer's technology, so automatic feature design has become an urgent demand for efficient image processing. Deep learning is a new research field of machine learning, which aims to study how to automatically extract multilevel feature representation from data. Its core idea is to extract multilevel and multiangle features from the original data by using a series of nonlinear transformations in a data-driven way, so that the obtained features have stronger generalization ability and expression ability. This just meets the needs of efficient image processing.

With the continuous development and deepening of financial markets, derivatives, economic forms, and systems, the knowledge, business models, and algorithms in the field of financial image technology analysis have become more and more professional and complex, and the corresponding large-scale data processing and analysis needs have also emerged. At the same time, people also began to use more information technology to assist financial data analysis. Among the information data analysis technologies, machine learning is the most popular one [1]. Machine learning is a subject in the field of artificial intelligence based on probability theory. In machine learning theory, there are many predictive image mathematical models and methods, among which deep learning theory has attracted much attention in recent two or three years. In recent years, artificial intelligence technology has developed rapidly. In terms of software and hardware, many technology manufacturers and academia around the world have made great progress in the field of artificial intelligence [2]. At the same time, there are many successful examples of deep learning applications in the industry, such as intelligent simultaneous translation system, face recognition, and semantic understanding. However, in the financial field, there are few examples of in-depth study. Deep learning belongs to machine learning and is also an important symbol of the development of artificial intelligence science to a certain stage [3]. In addition, the nonlinear characteristics of deep learning cater to a large number of random and uncertain factors in the financial market [4]. Therefore, based on the improved genetic multilayer neural network, under the background of deep learning theory and digital image economy, this paper discusses the applicability of intelligent model in the field of financial prediction, which has strong practical significance. In the second chapter, we first review the relevant literature of intelligent algorithms in the financial field, and then in the third chapter, we construct an improved genetic multilayer neural network algorithm based on the traditional neural network. In Chapter 4, the improved genetic multilayer neural network is applied to practical intelligent prediction and compared with the traditional neural network algorithm. The fifth chapter summarizes the full text.

## 2. Related Work

In recent years, artificial neural network has a great development, it has a strong nonlinear approximation ability, and it has a wide range of applications in intelligent control, classification and regression, image recognition, deep learning, and other fields. In particular, when combined with genetic algorithm, ant colony algorithm, and other optimization algorithms, artificial neural network shows infinite potential, which shows a new prospect for the research of natural science and social science. He et al. once built a neural network model to predict the daily return rate of IBM stock, based on which he built a short and long trading strategy. However, due to the gradient explosion problem, the prediction result of the trained model is not accurate, and the strategy effect is not very obvious [5]. Höpken et al. compared the prediction of time series between the neural network model and ARIMA model through experiments, and the results showed that the neural network had higher accuracy, but the paper did not discuss the improvement of the neural network model [6]. Avci et al. compared the Fama French three-factor model with the neural network model, and the related research showed that the prediction effect of the neural network model was better than the three-factor model [7]. Murat et al. developed the TOPIX prediction system to predict the weighted average index of the Tokyo Stock Exchange. The experimental results show that the prediction effect is ideal [8]. Peroni et al. used neural network model to forecast TCK stock market data, selected various types of parameters such as mode, volatility, power, and trend into the model, compared and analyzed the historical data, and finally proved that neural network is better in prediction and can be used to build portfolio [9]. Qu et al.'s research on neural network in the stock market involves more in the prediction of stock price and less in the prediction of stock index trend [10]. The selection of neural network model is more inclined to the classical model, the research of LSTM model is in the ascendant, and Koppe et al. studied the updated neural network models [11]. Hou et al. studied the improved feedback neural network. They proposed that there may be a large number of limit cycles or periodic motion solutions in the multilayer neural network of nonlinear dynamic system. In their research, not only is the pseudo attraction problem eliminated, but also the periodic solution which is often wasted is used, and good results are obtained [12]. Mikalef et al. used the dynamic characteristics of the feedback neural network to model the multi-input and output system. The results have very high stability and high accuracy and can also filter the external interference signal [13]. Roth et al. specially studied the dynamic characteristics of the feedback neural network, especially its stability, limit cycle, and chaos, and tested the optimization effect of the network, with good results [14]. Stergiou et al. studied the approximation ability of feedback neural network and found that it has strong approximation ability to nonlinear dynamic system, which greatly enriched the approximation theory of neural network [15]. In the aspect of stock forecasting, Mohammad et al. used artificial neural network to forecast the stock market. Since then, many scholars have been using various neural network models to try to simulate the stock market fluctuations. Both static neural network and dynamic neural network have achieved good results [16]. Kulisz et al. used fuzzy neural network to predict the time series of the stock market and genetic algorithm to optimize the investment strategy. Their research shows that the trend stock market is highly predictable, and the artificial neural network model combined with other artificial intelligence optimization methods can provide the accuracy of stock market prediction [17]. Wu et al. used multilayer feedforward BP neural network to predict the stock price and established the stock price prediction model based on the improved Elman neural network in their research. Taking the 180-day actual closing price of a single stock in Shenzhen stock market as the prediction object, the prediction accuracy, convergence speed, and stability of the experimental results are satisfactory [18]. Through the construction of different models and a large number of application studies, Ryotaro and Haruhiko have proved that neural network and other stock index prediction methods based on nonstatistical principles can better support the stock market prediction research [19]. But it also has some shortcomings. For example, there are many kinds of neural network models, each of which has its own unique advantages and disadvantages. It has different degrees of adaptability for the prediction research of the stock market. It still needs a lot of practice to choose which network is most suitable for this kind of application [20].

In addition, the number of hidden layers and image data transmission weights of neural network can only be selected according to the experience of researchers. Combining neural network with other artificial intelligence technologies, or improving and developing the existing image neural network to make it more effective and accurate investment prediction, is also a great challenge to the further research and application of artificial neural network prediction model. The PSO LSTM model is established, which is an improved and optimized model based on LSTM. It is good at dealing with complex nonlinear problems with long-term dependence. The PSO algorithm using adaptive learning strategy matches the characteristics of stock data with the network topology, which improves the accuracy of stock price prediction and the interpretability of model structure parameters. To sum up, many scholars have designed many prediction algorithms with good prediction effect and reference value in the field of stock price prediction, but there are also some problems: the first problem is that scholars focus on the improvement of the model itself, but have not developed effective feature selection technology; the second problem is that the combination of deep learning and integrated learning model is not used for financial price prediction.

## 3. Image Construction of Multilayer Neural Network Based on Improved Genetic Algorithm

### 3.1. Neuron Realization Perceptron

First, we need to introduce perceptron. In order to realize the artificial neural network, it is necessary to realize the basic unit neuron in the neural network. Perceptron can be regarded as neuron in artificial neural network. Neurons need computing power. Frank Rosenblatt defined the first perceptron with a single-layer computing unit in 1958. According to this definition, the learning signal of the perceptron is equal to the difference between the expected image output (teacher signal) and the actual output of the neuron, as shown in the following equation:

(1) $$\begin{array}{c} r=d_{j}-o_{j}, \end{array}$$

where *d*_*j*_ is the expected output, *o*_*j*_=*f*(*W*_*j*_^*T*^*X*) is the actual output, and the symbol function is the transformation function. The expression is as follows:

(2) $$\begin{array}{c} f\left(W_{j}^{T}X\right)=\mathrm{sgn}\left(W_{j}^{T}X\right)-\left\{\begin{array}{cc} 1, & W_{j}^{T}X\geq 0,\\ -1, & W_{j}^{T}X<0, \end{array}\right) \end{array}$$

where *X* is the input vector and *W*_*j*_^*T*^ is the weight vector at *T*. According to the above equation, the weight adjustment equation is derived as follows:

(3) $$\begin{array}{c} \Delta W_{j}=\eta \left[d_{j}-\mathrm{sgn}\left(W_{j}^{T}X\right)\right]X, \end{array}$$

(4) $$\begin{array}{c} \Delta W_{ij}=\eta \left[d_{j}-\mathrm{sgn}\left(W_{j}^{T}X\right)\right]x_{j},\;i=0,1,\ldots ,n, \end{array}$$

where *η* is the learning constant. When the actual output is the same as the expected output, the weight does not need to be adjusted. In the case of error, due to *d*_*j*_ and sgn(*W*_*j*_^*T*^*X*) ∈ {−1,1}, the equation of weight adjustment can be simplified as follows:

(5) $$\begin{array}{c} △W_{j}=\pm 2\eta X. \end{array}$$

Obviously, the learning rule of simple single-layer perceptron neural network is a linear function, and its weight is proportional to the input. This proportional relationship ultimately determines the functional relationship between network input and output. The final determination of function needs to constantly adjust the weight and threshold, which is a “training” process. The training process of neural network is also a learning process. In the process of neural network learning, the weights and thresholds of neurons at all levels will be adjusted according to certain methods and rules. These methods and rules involve image learning rules and training algorithms of neural networks. As a neuron in artificial neural network, the learning process of perceptron also follows the way of supervised learning. The learning rules of the perceptron can be summarized by the following simple equation:

(6) $$\begin{array}{c} e=t-a, \end{array}$$

where *t* represents the expected output value and *a* represents the actual output value. *e* is the difference between the expected output and the actual output, which represents the learning signal. When *e* is not zero, it means that the neural network needs further learning to make the expected output match the actual output. The positive and negative results of *e* determine the direction of adjusting the network weight threshold. When the expected output is greater than the actual output, the valves and channels in the network should be reduced or tightened. When the expected output is less than the actual output, the valves and channels in the network should be increased or released.

In neural networks, the transformation from input to output is called transfer function. The transfer function of the sensor is usually a threshold function, which makes the output of the network only 0 or 1. After a finite number of iterations, the ultimate goal of network training is to make the learning signal *e*=0*o* sensor need to obtain a batch of sample data input for training and learning. Each sample data is composed of a pair of input vectors and output vectors. The training sample set composed of *n* training samples is as follows:

(7) $$\begin{array}{c} \left\{p_{1},t_{1}\right\},\left\{p_{2},t_{2}\right\},\ldots ,\left\{p_{n},t_{n}\right\}. \end{array}$$

In the process of learning and training, the adjustment algorithm of the weight valve coefficient of each layer of neurons can be expressed by the following equations:

(8) $$\begin{array}{c} W\left(k+1\right)=W\left(k\right)+ep^{T}, \end{array}$$

(9) $$\begin{array}{c} b\left(k+1\right)=b\left(k\right)+e, \end{array}$$

where *e* is the error vector, *e*=*t* − *a*; *W* is the weight vector; *b* is the threshold vector; *p* is the input vector; *k* is the learning process of step *k*. Perceptron is the simplest neural network, corresponding to the neurons in biological neural network, which is the basis of studying and constructing other neural networks. Similarly, the learning rules of perceptron become the basis of learning rules of other more complex neural networks.

### 3.2. Improved Design of Multilayer Neural Network

**Figure 1:**
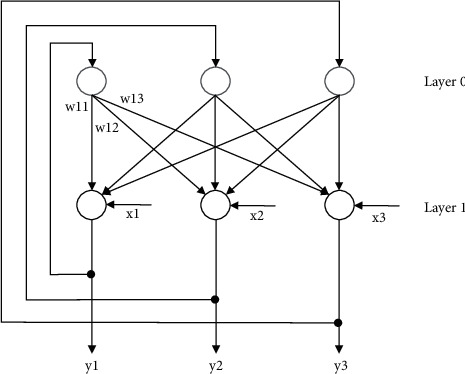
An improved multilayer neural network model.

This paper is improved on the basis of traditional genetic multilayer neural network. From its image network model, it is a recursive neural network. The feedback connection from output to input is shown in Figure 1. Although the number of neurons and layers of the network may not be large, due to the image structure characteristics of its cycle, in fact, it is a multilayer neural network. When running, the network depth should be determined by the number of cycles of input data in the middle layer of the network.

Under input excitation, the neuron units in the neural network will produce continuous state changes. For multilayer neural network, the key is to determine its image weight coefficient under stable conditions. The so-called stability condition refers to the introduction of a certain energy function into the multilayer neural network. When certain conditions are met, the energy will continue to decrease during the operation of the network and eventually tend to a stable equilibrium state. In fact, the condition that needs to be met is to constantly adjust the network weight. When the value of the energy function becomes smaller and smaller, the trend of the neural network to continue cyclic operation will weaken, so as to achieve the convergence effect. The energy in the multilayer neural network refers to the image scalar value associated with the current network state, which is expressed by the following equation:

(10) $$\begin{array}{c} E=-\frac{1}{2}\sum_{i.j}w_{ij}s_{i}s_{j}-\sum_{i}\theta_{i}s_{i}, \end{array}$$

where *w*_*ij*_ is the connection weight between the network node *i* and the node *j*; *s*_*i*_ and *s*_*j*_ are the states of node *i* and node *j*, respectively; *θ*_*i*_ is the threshold value of node *i*. The status update of the unit node is determined by the following equation:

(11) $$\begin{array}{c} s_{i}=\left\{\begin{array}{cc} 1, & \sum_{j}w_{ij}s_{j}\geq \theta_{i},\\ -1, & \text{other.} \end{array}\right) \end{array}$$

This equation shows the influence of the weight between two unit nodes on the state value of neurons. When the weight *w*_*ij*_ between the two points is greater than 0, when *s*_*j*_ is 1, the influence of neuron *j* on the total weight is positive, which drives the value of *s*_*i*_ to 1. When *s*_*j*_ is -1, the effect of *j* on the total weight is negative, which drives the value of *s*_*i*_ to -1. Therefore, when the weights between neurons *i* and *j* are positive, their values will converge, and when the weights between neurons *i* and *j* are negative, their values will diverge. The “energy” equation in multilayer neural network ensures that the energy value either decreases or remains unchanged when the neuron units are randomly selected and updated. After continuous state updating, the network will eventually converge to the state of the local minimum in the energy function (which is considered as Lyapunov function). Therefore, when the state is a local minimum in the energy function, the network reaches a stable state.

## 4. Empirical Analysis of Forecasting

### 4.1. Data Sample Introduction

This experiment will use the data of northern rare earth (600111) stock in recent five years (from September 2013 to August 2018) to verify the research. As can be seen from the figure below, the trend of the stock has shown some fluctuations in the past five years, with two obvious upward trends. The stock cycle form is more abundant, which is more conducive to do research. In the five-year data, the data of the first three years (September 2013 to August 2016) will be used to build the model and its parameters. The data for the next two years (September 2016 to August 2018) will be used to verify the efficiency of the model.

### 4.2. Improved Multilayer Neural Network Prediction Based on Technical Indicators

This experiment will use genetic multilayer neural network for prediction. The training data is the historical closing price from September 2, 2013 to August 31, 2016, and the genetic multilayer neural network is trained. After comprehensive training, the final network structure and its parameters are determined. The number of hidden layers is the number of hidden layers (middle layers) in shennet. The number of hidden layers directly determines the learning depth of neural network. In theory, the more the hidden layers, the higher the accuracy of the network algorithm, the better the learning effect.

**Figure 2:**
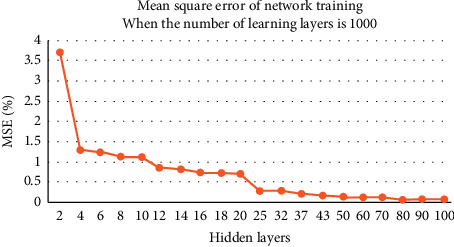
Network error when the number of learning times is 1000.

Learning times refer to the learning times of neural network in unified input. For each learning, the neural network will adjust the weight threshold to make the output into the actual results. In theory, the more the times of learning are, the better the network is trained, and the accuracy of network calculation can be improved. This experiment will train the network through the different combination of the number of hidden layers and the number of learning times. The learning results are evaluated by the mean square error of network training, and finally the appropriate combination to build the verification network is found. The number of learning times is fixed at 1000, and 20 hidden layers are combined to test. The experimental results are shown in Figure 2.

**Figure 3:**
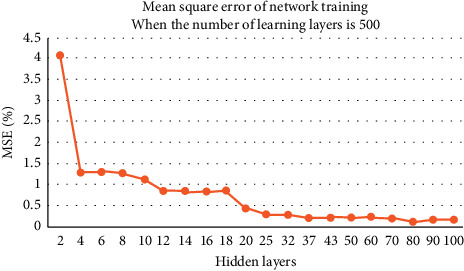
Network error when the number of learning times is 500.

The number of learning times is fixed at 500, and 20 hidden layers are combined to test. The experimental results are shown in Figure 3.

**Figure 4:**
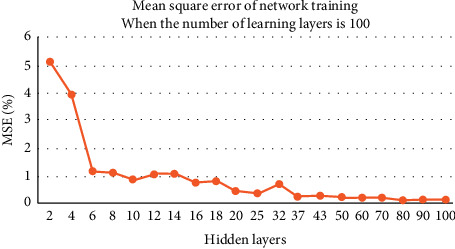
Network error when the number of learning times is 100.

The number of learning times is fixed to 100, and 20 hidden layers are combined to test. The experimental results are shown in Figure 4.

The above three groups of image experiments show that, with the increase of the number of hidden layers, the network calculation error decreases and the accuracy improves. For the image scene with 1000 learning times, when the number of hidden layers reaches 80, the error rate can be reduced to less than one thousandth. For 500 and 100 learning image scenes, when the number of hidden layers reaches 100, the error rate is not less than one thousandth.

In order to achieve the same error standard, more experiments show that, for the image scene with 500 learning times, when the number of hidden layers reaches 120, the network bit error rate can be reduced to less than one thousandth. For the image scene with 100 learning times, when the number of hidden layers reaches 150, the network error rate can be reduced to less than one thousandth. Although the number of hidden layers can be found for each learning time, which makes the network calculation error less than one thousandth, and achieves the same effect, the calculation time consumed by each learning time and hidden layer combination is obviously different.

Therefore, after synthesizing the performance comparison of network calculation error and calculation time, this experiment will use the third set of network parameters in the above table to build the verification network.

**Figure 5:**
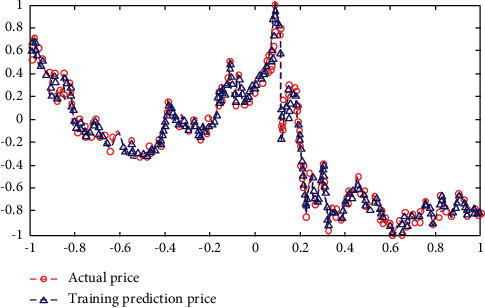
Simulation of the data from September 20, 2013, to August 31, 2016.

Experimental results in Figure 5 show that the simulation results of the network are highly consistent with the historical real data from September 2, 2013, to August 31, 2016.

**Figure 6:**
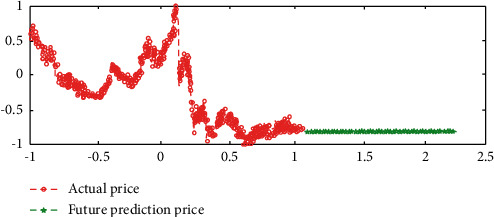
The predictions of the data from September 1, 2016, to August 31, 2018.

The experimental results in Figure 6 show that the network obtained by training can not judge the future data trend at all. This experiment is based on the stock's own historical price information to judge the future price trend. Although the simulation efficiency of the network is very high, the prediction result is completely out of control. However, we can deeply understand the following characteristics of deep learning and neural network from the results: it is difficult to get the desired prediction results using single, isolated, and unprocessed sample data. Although the data result of network simulation is very successful, there is no effective mapping relationship from input data to output data in network training. This experiment is actually a simulation based on time series, but if the periodicity of the series is not found, the trained network and its function will be meaningless.

### 4.3. Prediction Based on Technical Index of Improved Multilayer Neural Network

Because the multilayer neural network is a self cyclic neural image network, and the number of cycles is determined by the properties of energy function and input data, it is not necessary to set similar parameters in the process of constructing the multilayer neural network, such as the number of learning times and the number of images in the hidden layer. MACD index is used as input data for network training. The combination of MACD index and KDJ index is used as the input data of network training. EDMA function is required for calculation. The details from (12) to (15) are as follows:

(12) $$\begin{array}{c} {\text{RSV}}_{t}=100\times \frac{\left({\text{close}}_{{\text{price}}_{t}}-\text{LLVD bottom}\_{\text{price}}_{t},\text{period}1\right)}{\left(\text{HHV}\left({\text{top}}_{{\text{price}}_{t}},\text{period}1\right)-\text{LLV}\left(\text{bottom}\_{\text{price}}_{t},\text{period}1\right)\right)}, \end{array}$$

(13) $$\begin{array}{c} K_{t}=\text{EDMA}\left({\text{RSV}}_{t},\text{period}2,1\right), \end{array}$$

(14) $$\begin{array}{c} D_{t}=\text{EDMA}\left(K_{t},\text{period}3,1\right), \end{array}$$

(15) $$\begin{array}{c} I_{t}=3K_{t}-2D_{t}. \end{array}$$

**Figure 7:**
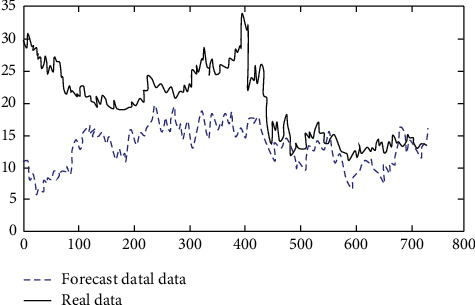
A predictive effect based on a multilayer neural network when the number of layers is 7.

**Figure 8:**
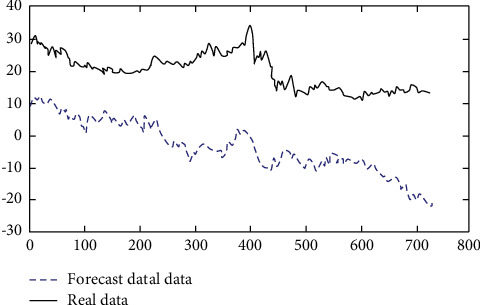
A predictive effect based on a multilayer neural network when the number of layers is 5.

**Figure 9:**
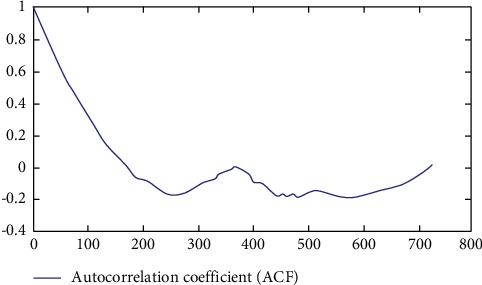
Autocorrelation coefficient of the closing price of northern rare earth from September 2013 to August 2016.

It can be seen from Figures 7 and 8 that when the number of layers is 5, the simulation is greatly distorted, and when the number of layers is 7, it is relatively close to the real data, and the judgment of the operation direction is consistent in many cases. Therefore, after the above experiments, the number of layers is set as 5. The autocorrelation coefficient and partial autocorrelation coefficient of the sample are calculated.  Figure 9 shows all historical graphs of autocorrelation coefficient and partial autocorrelation coefficient.

**Figure 10:**
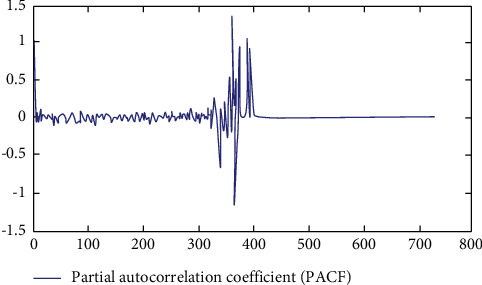
The closing price of the northern rare earth varies from September 2013 to August 2016.

**Figure 11:**
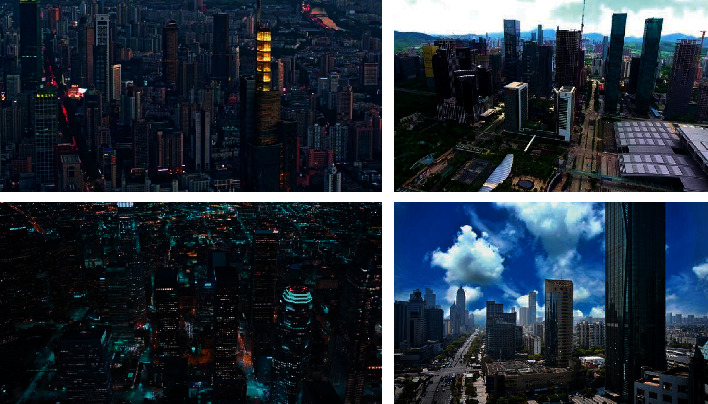
Urban changes and opportunities brought by the transformation of digital economy.

Combined with the description of stationary condition, it can be seen from Figure 10 that the autocorrelation coefficient graph and partial autocorrelation coefficient graph of the time series have obvious tailing phenomenon from the beginning. The partial autocorrelation coefficient approaches to 0 rapidly, and the autocorrelation coefficient tends to 0 rapidly. In the model validation part, the historical closing price from September 1, 2016, to August 31, 2018, will be used as the input data, and the prediction data generated by the improved multilayer neural network model will be compared. From the above test patterns and error rate, it can be seen that 7 layers is the most successful prediction, but the prediction success rate reaches 77.83%. Take the data of one month from August 1 to 31, 2018, when the number of floors is 7 as an example. From Table 1, we can see that the judgment of operation direction is consistent in many cases, and the prediction efficiency is very high. Opportunities brought by the new round of digital economy reform, as shown in Figure 11.

## 5. Conclusion

Deep learning is a kind of machine learning, but it is different from the traditional machine learning method. It uses the deep (multilayer) image neural network structure, including the network hierarchy and the connection rules between neurons, to connect the algorithms of each image neuron in the network to form a knowledge system that can understand, express, and even predict the objective world. This knowledge system is difficult to express by laws or equations. It is similar to the structure and learning mode of human brain. This way of learning and understanding the world only avoids the general thinking caused by many theoretical assumptions, but covers all fait accomplis. When predicting the future, the trained neural network will select the path to be calculated according to the image rules (weight threshold algorithm, etc.) formed in the network and finally output the answer. Compared with traditional multilayer neural network, the calculation speed of multilayer neural network is very fast. For the same amount of input data, traditional multilayer neural network only takes a few minutes and genetic multilayer neural network only takes a second. However, the fast convergence ability of multilayer neural network also limits the time to complete its own network algorithm, resulting in a large error between the output results and the actual results. The highest prediction ability of the three experiments is only 60.87%. In multiple tests of the same scene, the multilayer neural network shows the consistency of the results, and there will be no different prediction results. Although the results of all multilayer neural network prediction experiments are worse than the image results of BP network prediction experiments, the multilayer neural network also reflects the trend that the prediction success rate increases with the increase of the dimension of input data. This shows that, by adding more technical indicators as the image input of the combined neural network, the prediction efficiency of the multilayer neural network can be further improved and the advantage of computing speed can be maintained.

## Figures and Tables

**Table 1:** Data forecast for one month from August 1 to 31, 2018, when the number of layers is 7.

Date	Real data			Forecast data			
	Price	Amplitude	Ups and downs	Price	Amplitude	Ups and downs	Match real ups and downs
2018/8/1	10.83	NA	−1	9.44	NA	−1	Yes
2018/8/2	10.58	−2%	−1	9.70	3%	1	No
2018/8/3	10.51	−1%	−1	9.62	−1%	−1	Yes
2018/8/6	10.29	−2%	−1	9.57	−1%	−1	Yes
2018/8/7	10.6	3%	1	9.44	−1%	−1	No
2018/8/8	10.44	−2%	−1	9.54	1%	1	No
2018/8/9	10.61	2%	1	9.59	0%	1	Yes
2018/8/10	10.58	0%	−1	9.51	−1%	−1	Yes
2018/8/13	10.5	−1%	−1	9.33	−2%	−1	Yes
2018/8/14	10.63	1%	1	9.30	0%	−1	No
2018/8/15	10.43	−2%	−1	8.93	−4%	−1	Yes
2018/8/16	10.31	−1%	−1	8.99	1%	1	No
2018/8/17	10.18	−1%	−1	9.09	1%	1	No
2018/8/20	10.22	0%	1	9.01	−1%	−1	No
2018/8/21	10.26	0%	−1	9.26	3%	1	Yes
2018/8/22	10.1	−2%	−1	9.47	2%	1	No
2018/8/23	10.1	0%	0	9.62	2%	1	No
2018/8/24	10.16	1%	1	9.42	−2%	−1	No
2018/8/27	10.31	1%	1	9.96	6%	1	Yes
2018/8/28	10.4	1%	1	10.09	1%	1	Yes
2018/8/29	10.26	−1%	−1	9.84	−3%	−1	Yes
2018/8/30	10.16	−1%	−1	10.12	3%	1	No
2018/8/31	10.24	1%	1	9.64	5%	−1	No

## Data Availability

The data used to support the findings of this study are available from the corresponding author upon request.
